# “She’s gone now.” A mixed methods analysis of the experiences and perceptions around the deaths of children who died unexpectedly in health care facilities in Cape Town, South Africa

**DOI:** 10.1371/journal.pone.0213455

**Published:** 2019-03-06

**Authors:** Peter Hodkinson, Jessica Price, Caroline Croxson, Lee Wallis, Alison Ward, Andrew Argent, Stephen Reid

**Affiliations:** 1 Division of Emergency Medicine, University of Cape Town, Cape Town, South Africa; 2 Nuffield Department of Primary Care Health Sciences, University of Oxford, Oxford, United Kingdom; 3 Department of Paediatrics University of Cape Town, Cape Town, South Africa; 4 Directorate of Primary Health Care, University of Cape Town, Cape Town, South Africa; Harvard Medical School, UNITED STATES

## Abstract

**Purpose:**

The sudden death of a child is a catastrophic event for both the family and the healthcare workers involved. Confidential enquiries provide a biomedical depiction of the processes and quality of care delivered and drive improvements in care. However, these rarely include an assessment of the patient/caregiver experience which is increasingly regarded as a key measure of quality of care.

**Methods:**

A parallel convergent mixed methods design was used to compare and contrast medically-assessed clinical quality of care with caregiver perceptions of quality and care in a cohort of sudden childhood deaths in emergency facilities in Cape Town, South Africa.

**Results:**

Amongst the 29 sudden childhood deaths, clinical quality of care was assessed as poor in 11 (38%) and the death was considered avoidable or potentially avoidable in 16 (55%). The main themes identified from the caregivers were their perception of the quality of care delivered (driven by perceived healthcare worker effort, empathy and promptness), the way the family was dealt with during the final resuscitation, and communications at the time of and after the death. Ten (35%) caregivers were predominantly negative about the care delivered, of whom four received fair clinical quality of care; 13 (49%) of caregivers had predominantly positive experiences, one of whom received poor clinical quality of care.

**Conclusions:**

Caregivers’ experiences of the healthcare service around their child’s death are influenced largely by the way healthcare workers communicate with them, as well as the perceived clinical effort. This is not always concordant with the clinically assessed quality of care. Simple interventions such as protocols and education of healthcare workers in dealing with families of a dying or deceased child could improve families’ experiences at a time when they are most vulnerable.

## Introduction

Despite significant improvements in the last decade, South Africa failed to meet the Millennium Development Goals relating to neonatal and under-5 mortality [1]. Neonatal conditions, respiratory tract infection, gastro-enteritis and malnutrition continue to account for the majority of deaths, with HIV being an important co-morbidity. In older children, trauma and unnatural causes of death are also common [2]. Confidential enquiries are a well-established method to investigate child deaths, and have been used in South Africa since the late 1990’s to identify modifiable factors that contributed to death [3]. This methodology has been shown to improve outcomes in high as well as low-resource settings [4, 5]. However, these methods typically focus on a biomedical approach to assessment of quality of care and avoidability of adverse events, with findings commenting on cause of death, age group, and points of delay from the perspective of healthcare workers. Generally, such methods do not attempt to assess the experience or perceptions of care of the family of the deceased.

Increasingly the quality delivered by a healthcare system is understood to include the patient experience, along with patient safety and clinical effectiveness [6]. A systematic review of the links between these quality measures, all in high income countries, showed consistent positive associations–meaning that we need to take patient experience seriously [7]. Reports of patient experience in lower income settings such as South Africa are scarce, and largely limited to specific long term treatment outcomes and satisfaction with TB and HIV management [8, 9], yet the patient experience is well recognized as a priority [10, 11].

Understanding and monitoring the determinants of patient experience is increasingly a focus of quality of care assessment [12]. Such assessment is particularly important at times when patients and their families are most vulnerable. When children die, it becomes important to understand the experience of their families, and particularly those of the caregivers (usually the parents). Priorities of parents during end-of-life care from a US study [13] were shown to include regular and honest communication, supportive staff and preserving the parent-child relationship. However, their experiences are also difficult to capture: the final resuscitation, the management of the death itself, and management of the family is emotive. Death in the emergency context is usually sudden and unexpected, making evaluation of the experience very difficult; further, the experience of death (particularly that of a child) is a personal, and potentially very private experience so accessing that information can only be done with great sensitivity and with the trust of the people concerned.

As part of a larger study involving caregivers of critically ill and injured children in Cape Town, South Africa [14], interviews were conducted with caregivers whose children died in healthcare facilities; together with the medical records of these deaths, we are able to put together a detailed analysis of the care during the child’s death. The aim of this study was to identify practices which influenced the caregivers’ experience and satisfaction with the care of their child around the time of death, as well as to compare caregivers’ experience with medical assessment of care quality.

## Methods

### Setting and participants

This study used mixed methods to evaluate the quality of care and the experiences of healthcare of the caregivers of a cohort of children who died in health facilities in the Cape Town Metro area. Data were drawn from the Pathways to Care Project, a study conducted from the main tertiary paediatric referral hospital in Cape Town, the Red Cross War Memorial Children’s Hospital (RCWMCH) over 12 months from November 2011 to October 2012 [14]. This study used an extension of the child death review (confidential enquiry) concept which is well described elsewhere [1, 2, 15, 16]. Cases of critically ill and injured children under 13 years old were reviewed to uncover the entire health care referral pathway for their critical event, by collecting health records from every facility involved in the acute care pathway, interviewing the caregiver of each child, and conducting an expert review for each child. Study participants were recruited from two sources: the majority were critically ill and injured children admitted to the RCWMCH Paediatric Intensive Care Unit (PICU) with an emergency healthcare episode–these children were sampled by alternate week recruitment throughout the year-long study; the second group were children who died suddenly in healthcare facilities in the RCWMCH metropolitan drainage area, or who died in RCWMCH Emergency Centre. This study has been well described in two publications, one focused primarily on the quality of clinical care along the care pathway, looking specifically for modifiable factors and avoidable events [14], and the other a qualitative paper with a focus on the experiences of caregivers in seeking care for their children [17].

Neither of the prior publications focused on the cohort of children who died within the healthcare services prior to PICU admission, which is the focus of this paper. We excluded children without a full interview with the caregivers, those with chronic medical conditions, those admitted to any hospital for more than 5 days prior to death, deaths following a palliative care decision, neonates direct from obstetric services, and those without signs of life on arrival at the healthcare facility. All eligible deaths documented over the year-long period were included. Semi-structured in-depth interviews were conducted by one of three trained interviewers within two weeks of the death of the child. All interviews were conducted in the home language of each caregiver, recorded, transcribed and then translated into English by the interviewer. An interview guide developed by the authors and refined after a pilot of 20 caregivers, was used to draw details on the referral pathway, timeline and the caregivers’ perceptions and observations on the care received (S1 Table), described in detail by Jones et al. [17]. All data were collected with the intention of using mixed methods to analyse and understand the two datasets together–representing essentially a clinical, consensus-based assessment, as well as the caregivers’ observations and perceptions of the care. Mixed methods analysis enables deeper understanding than qualitative or quantitative analysis alone, and is a useful tool to enhance the value of a dataset, and to explore deeper issues around a phenomenon such as patient experience [18].

### Data analysis

#### Quantitative analysis

Quantitative data included quality of care (QOC) as assessed by consensus of an expert panel, following detailed review of the medical records and caregiver interview for each child. The expert panel comprised a paediatric intensivist, emergency physician and a primary health care expert, all senior clinicians in this health system, but none had dealt directly with the patients described. The primary assessments of QOC were: a) the avoidability of death by interventions in the final acute care pathway (*avoidable*, *potentially avoidable* or *not avoidable*); and b) global QOC across the referral pathway (*good*, *fair* or *poor*) which looked at the overall care delivered in the acute pathway (this often involved several facilities/ambulances, with potentially variable individually assessed QOC). These assessments were primarily made from a clinician’s point of view, although with the insights of the caregivers’ experience, and represent a predominantly biomedical assessment of the QOC. Further details are published elsewhere [14].

#### Qualitative analysis

Qualitative data analysis was based on grounded theory using an inductive approach to the analysis. Thematic analysis of the caregiver interviews was conducted. Two researchers (PH & JP) read all the transcripts for children who had died prior to PICU, and together developed a coding scheme. Both researchers coded all the data, and checked one-another, deliberating any contentious issues. Both PH and JP are clinicians with experience in the Cape Town healthcare system (one with in depth insights to the study and data collection (PH), and the other naïve to the quantitative outcomes (JP)). The coding scheme and emergent themes were discussed with other authors (AA & SR), reassessed and simplified into several coding matrices to represent the [19] key issues identified. Cases were searched for confirmatory as well as outlier material. Following immersion and analysis of each interview, an overall judgement was made by the two primary investigators (PH & JP) as to the caregivers’ expressed level of satisfaction with the healthcare delivered to each child (positive, neutral or negative).

#### Mixed methods analysis

**Fig 1:**
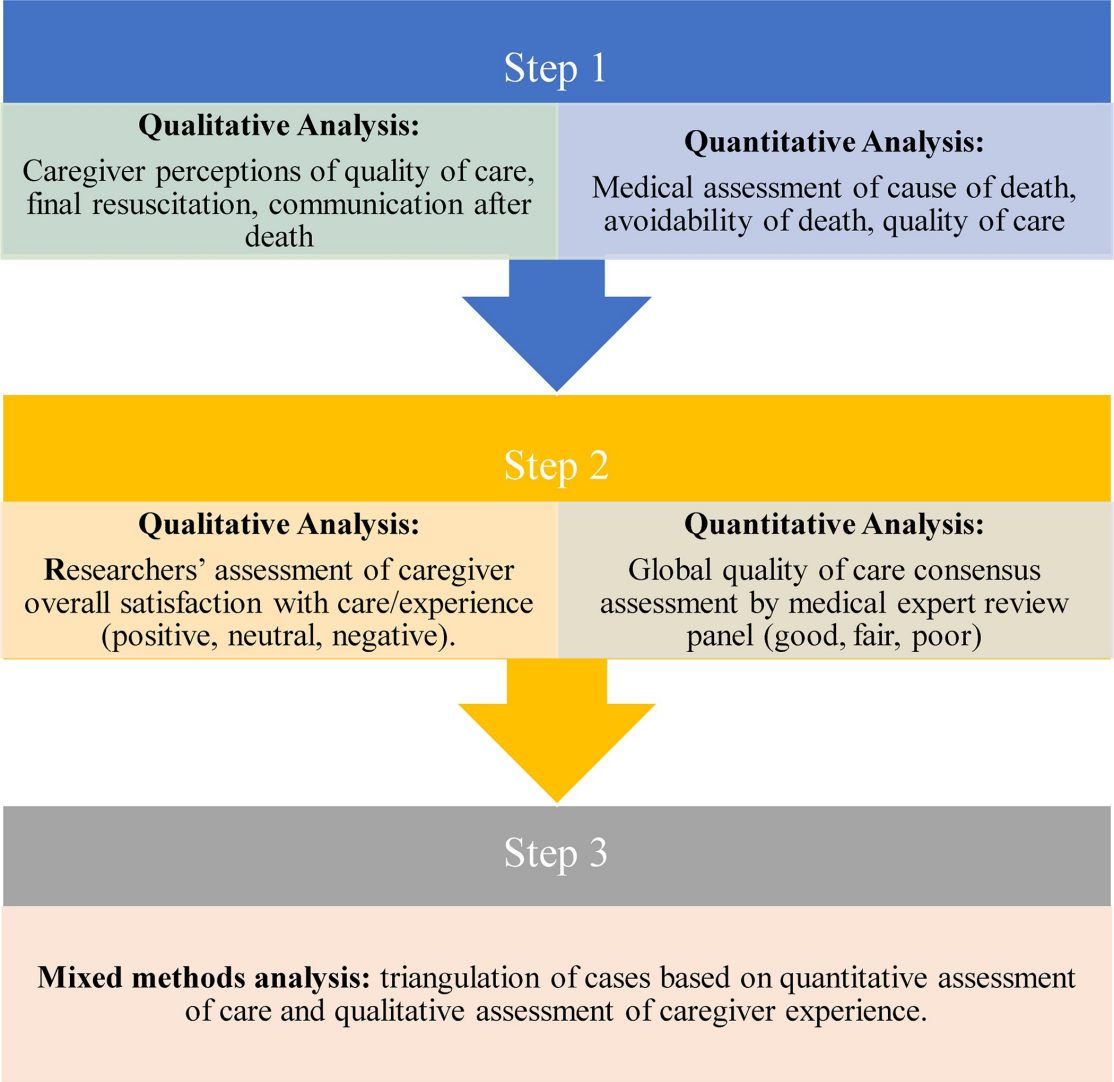
Mixed methods approach to analysis.

A convergent, parallel mixed methods design was used, with quantitative and qualitative data collected and analysed separately, and findings then triangulated using a mixed methods matrix (Fig 1) [19]. Equal priority was given to qualitative and quantitative data. The matrix allowed themes and categories from both analyses to be visualized and compared for each case, and overall, to integrate the findings, and to search for patterns and meaning.

#### Ethical considerations

The study was approved by the University of Cape Town Human Research Ethics Committee (HREC 211/2011), by the Oxford Tropical Research Ethics Committee, Oxford University (OXTREC 29–11), and site approvals to collect data were obtained from the Western Cape Department of Health and the City of Cape Town. Caregivers gave written consent to participate and for their interviews to be recorded and transcribed.

## Results

### Quantitative analysis

Over the 12-month study, there were 29 children who died prior to PICU and all were included. The demographics of these children and their caregivers (mothers were interviewed in all cases, with fathers also present for 2 interviews) are shown in Table 1.

**Table 1:** Demographics of children and families.

**CHILDREN’S CHARACTERISTICS**		n	(%)
Gender	Male	19	65.5
	Female	10	34.5
Age	<1 month	1	3.4
	1 month– 1 year	16	55.2
	1-5 years	5	17.2
	>5 years	7	24.1
**MOTHERS’ CHARACTERISTICS **			
Employment	Full time	12	41.4
	Part time	2	6.9
	Unemployed	15	51.7
Maternal Age	≤ 21 years	1	3.4
	22–34 years	18	62.1
	>34 years	10	34.5
Marital Status	Single	15	51.7
	Married/ long term partner	14	48.3
Education	Grade 8 or less	8	27.6
	Grade 9/10	14	48.3
	Grade 11/12/ tertiary	7	24.1
Dwelling Type	Informal	10	34.5
	Formal on separate stand	10	34.5
	Flat/ townhouse	9	31.0
Language	Xhosa	15	51.7
	Afrikaans	13	44.8
	Other	1	3.4
Household Income/month	< R1000	11	37.9
	R1000-2500	10	34.5
	>R2500	8	27.6
Distance to nearest 24-hour facility	<5 km	19	65.5
	>5 km	10	34.5
Transport to nearest 24 hr facility	Taxi	18	62.1
	Walk	6	20.7
	Private Car	5	17.2

There were 15 deaths at healthcare facilities prior to arrival at RCWMCH, and 14 deaths in the Emergency Centre of RCWMCH (Table 2). Ages of children at time of death were similar in both groups (mean age of deaths prior to RCWMCH 34 months; IQR 4, 37 and mean age of deaths at RCWMCH 40 months; IQR 8, 70). Infection was the likely aetiology of the deaths in a third of cases (10, 34.5%), and just over a quarter died due to trauma (8, 27.6%). The reported delay from onset of illness to first presentation for these cases was less than a day for 19 (65.5%) cases, suggesting a rapid (or instant for trauma) disease progression. More than three quarters presented after hours. All had a brief pathway, with 18 (62.1%) visiting only the facility where they died, and only 12 (41.4%) being transferred by ambulance. There was no clear correlation between child’s age at death and any of the outcome measures.

**Table 2:** Description and outcomes of the cohort.

		Deaths prior to RCWMCH (n = 15)		RCWMCH EC Deaths (n = 14)		All deaths prior to PICU (n = 29)	
Medical or Trauma	Medical	13	86.7%	8	57.1%	21	72.4%
	Trauma	2	13.3%	6	42.9%	8	27.6%
Cause of Death	Cardiac	0	0.0%	3	21.4%	3	10.3%
	Gastro-enteritis	2	13.3%	0	0.0%	2	6.9%
	Respiratory	2	13.3%	3	21.4%	5	17.2%
	Sepsis	2	13.3%	2	14.3%	4	13.8%
	Trauma	2	13.3%	6	42.9%	8	27.6%
	Unknown	7	46.7%	0	0.0%	7	24.1%
Global Quality of Care	Poor	7	46.7%	6	42.9%	11	37.9%
	Fair	6	40.0%	7	50.0%	13	44.8%
	Good	2	13.3%	3	21.4%	5	17.2%
Avoidability of death	Not Avoidable	5	33.3%	8	57.1%	13	44.8%
	Potentially Avoidable	8	53.3%	5	35.7%	13	44.8%
	Avoidable	2	13.3%	1	7.1%	3	10.3%
Duration from onset illness to first presentation	< 1 day	10	66.7%	9	64.3%	19	65.5%
	1–3 days	1	6.7%	2	14.3%	3	10.3%
	> 3 days	4	26.7%	3	21.4%	7	24.1%
Time of first presentation	Office Hours (8–16)	4	26.7%	2	14.3%	6	20.7%
	After Hours (16–8 & weekends)	11	73.3%	12	85.7%	23	79.3%

RCWMCH, Red Cross War Memorial Children’s Hospital; EC, Emergency Centre; PICU, Paediatric Intensive Care Unit.

Following an expert panel confidential enquiry approach on each case, it was considered that death was *avoidable* or *potentially avoidable* in more than half of cases (16, 55.2%) and global QOC was considered *poor* in 37.9%. The major modifiable factors identified in this cohort using categories as described in the prior quantitative publication [14], included: (a) ***initial assessment***: inadequate assessment or interpretation of severity, missing key clinical findings; (b) ***management***: resuscitation not done or inadequate for shocked patient, circulatory issues, delay in critical management decisions, referral delay; (c) ***ambulance services***: response time delay). Poor communication with caregivers was also identified as a modifiable factor in many cases.

### Qualitative results

**Fig 2:**
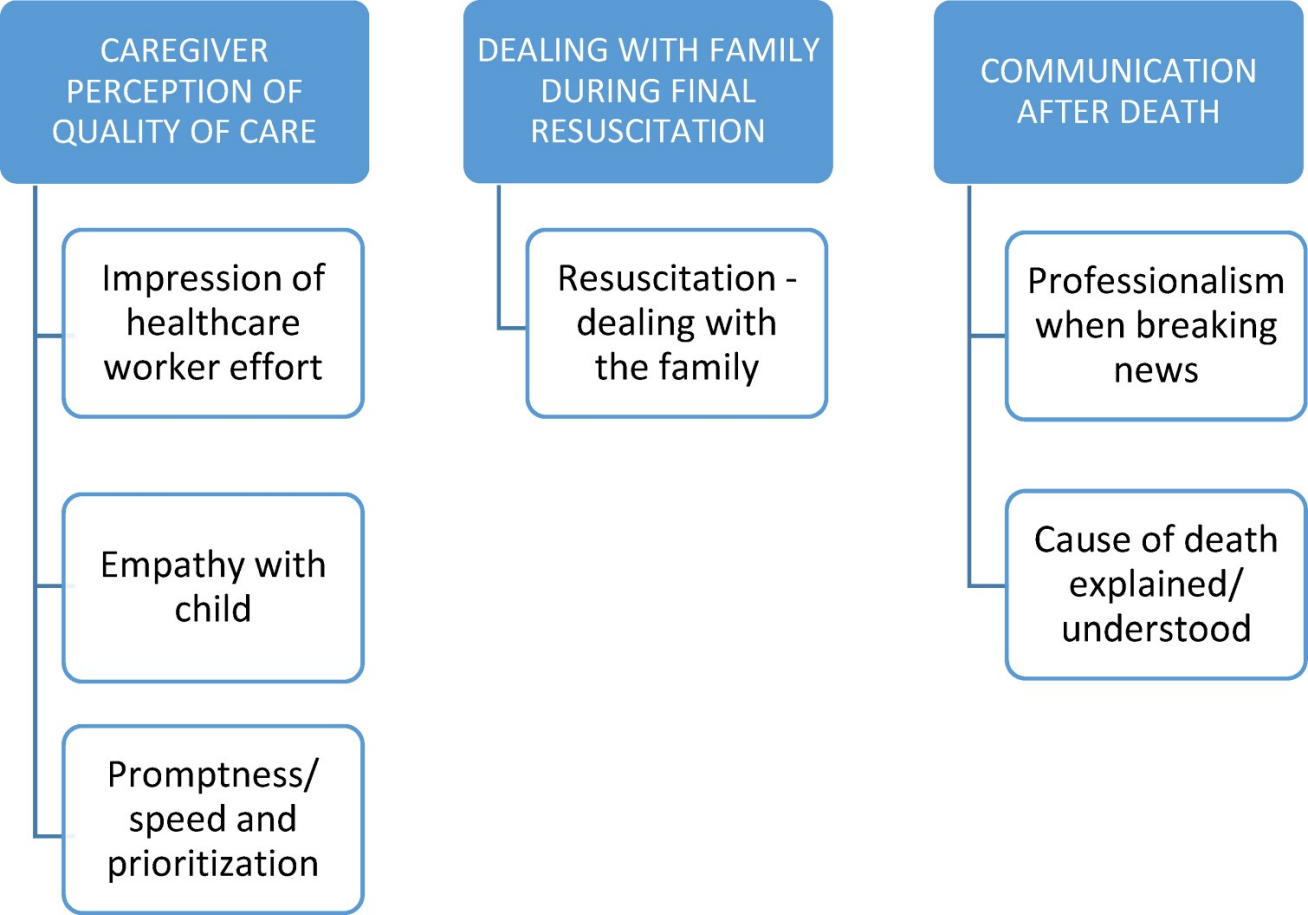
Qualitative themes developed from caregivers’ perceptions.

Regarding caregivers’ experiences of the healthcare system and their satisfaction with the care, three main themes emerged. The themes and subthemes are depicted in Fig 2 and discussed below. An overall assessment of the caregiver impression of the care received showed 13 caregivers to be positive, six neutral, and 10 negative about the care that they had received.

*(Annotations to each quote refer to the various quality assessments viz*. *[panel global assessment of care (GOOD/FAIR/POOR); panel assessment of avoidability of death (AVOIDABLE (AVOID)/POTENTIALLY AVOIDABLE (PA)/NOT AVOIDABLE (NA))*; *and qualitative overall assessment of caregiver’s satisfaction (Positive/ Neutral/ Negative)])*

#### Impression of healthcare worker effort

Caregivers’ perception of healthcare worker effort was very mixed: impression of effort seemed related to both what was done (visible procedures e.g. putting up drips, giving antibiotics, connecting to cardiac monitors etc.), the number of people called to work on the child and whether they were taken away from other patients to attend to their child:

*I think they tried all they can to help my child*, *I have no complaints because I saw all the machines that were used to assist my child and she had drips put in her body and needles so I don’t think there was anything else that was supposed to be done to save her*. *[FAIR; PA; Positive*]

…*they tried everything to help him–honestly*, *I can tell you they tried their best* … *They struggled with him* …, *and I stood there and I sat there*. *Four doctors–those doctors actually pushed the other mothers with their sick babies aside and they struggled (worked) with him [POOR; PA; Neutral]*

*They left all their other work*, *just to work with her*…. *The doctor talked to me*, *yes*. *Whenever they had finished working with her*, *he would explain what they just did*. *The ambulance trip was… Their service was good because whenever something was wrong*, *they would pull over and then… they tried their best–they did*. *From what I saw*, *they were just busy with her the whole time*. *They brought their other work to a standstill*, *just to work with her*. *[FAIR; PA; Positive]*

*They tried everything and then they*… *because they did a lot of CPR*.*… They left their other patients and came and helped me*. *No*, *their service was very excellent and very professional at the hospital*. *[FAIR; NA; Positive]*

#### Empathy with child

The perceived empathy of professionals was appreciated.

*And they were very sympathetic and you could see*, *it was actually… they tried their utmost and they were very disappointed that they couldn’t like revive her*. *[FAIR; NA; Positive*]

However, in some cases, caregivers articulated a lack of empathy and professionalism:

*I told her this child is struggling to breathe; she didn’t do nothing… she (nurse) said*, *“Oh*, *this child was so sick*. *I was supposed to do something*.*” But she didn’t*. *She didn’t help my child*. *What was she doing all that time*?! *What was she busy with*?! *But when she came to me*, *then I saw what she was busy with*. *She was more on her phone than anything else*! *[POOR; AVOID; Negative*]

There were also examples of insensitive and uncaring attitudes from healthcare workers.

*I struggled with her… with the drip because I couldn’t (manoeuvre) the pan… And they were shouting at me and the foam came (out of her mouth) … I held her because I wanted to put her back on the bed and they didn’t want me to… she wasn’t off the bed*, *I just lifted her up*. *And they were shouting at me*. *[POOR; PA; Negative*]

*I was telling her*, *“You can see the boy is sick*.*” But she just told me… so*, *she called security–he must just chuck me out*. *[POOR; AVOID; Negative*]

#### Promptness/Delays in being attended to or transferred

In general, caregivers were satisfied that children were attended to promptly on arrival. All the trauma cases were seen very rapidly at facilities, and for medical cases generally, these children arrived in extremis and were seen rapidly, with some exceptions.

*I will not lie*, *I am very happy with the care my child received from the doctor*. *He immediately attended to him on arrival*. *[POOR; AVOID; Neutral*]

..*and when I asked the security (guard)*: *“What’s going on here*? *They’re standing there and nobody seems to want to help me*.*” So*, *he went in…so*, *he went to tell them that there was a patient in front… waiting outside*. *[POOR; NOT AVOID; Negative*]

*It’s crazy (deurmekaar) over there*. *If you get there after that person and your child is serious*, *you just have to wait for your name to be called even though they can see the child is serious*. *[POOR; PA; Neutral*]

Ambulance transfers were a source of delay and impacted negatively on the perception of care, sometimes even when services at healthcare facilities were well regarded.

*And I waited until I couldn’t anymore*. *I started to panic because his eyes were getting big*. *“Sister when…” “No*, *XXX*, *the ambulance is on its way*.*” Every time it’s*, *“No*, *XXX the ambulance is on its way*.*” I even called the doctor–(and asked him) “Doctor*, *what now*?*” “Uhm*, *XXX*, *we have to wait for the ambulance*.*” Oh*, *gosh*, *we waited and waited and eventually they arrived*. *[POOR; PA; Neutral*]

Many caregivers in Cape Town perceive referral to the tertiary paediatric hospital, RCWMCH as the panacea for any critically ill child and often questioned why their child wasn’t referred. In most cases the experiences were positive at RCWMCH, but not always.

*I said*, *“Can’t you refer me to Red Cross (Hospital) please*?*” …*.. *If only I’d driven past the Day Hospital and gone straight to Red Cross (Hospital)* … *I think they could’ve sent me to Red Cross (Hospital) since his temperature hadn’t gone down*. *[POOR; PA; Positive*]

*Why didn’t they say*, *“Mummy*, *we’re going to send the child to Red Cross now*,*” or whatever*? *She still qualified to go to Red Cross; she was 12 years old*. *[POOR; AVOID; Negative*]

*We had so much faith in Red Cross but if I knew what I know now*, *I would’ve taken him out of there and taken him to another hospital… a private hospital and then we would’ve just had to pay the… But because Red Cross has got this fantastic reputation*, *we thought there’s no better place for the child to be*. *[POOR; AVOID; Negative*]

#### Dealing with the family during the final resuscitation attempt

Most caregivers (22, 88.0%) expressed the desire to be present during the resus, though few (8, 32.0%) were permitted to be there.

*I wanted to see what they were doing but then they said I must wait outside they will tell me [FAIR; PA; Positive*]

*I was asked to wait outside…*.. *as a mother you would want to be there for your child in a such a time of need but I did understand that doctors have to do their job*. *[FAIR; NA; Positive*]

*Whilst still waiting outside I also sneaked and went to peep but my baby was concealed by a curtain and could not see what was happening*. *I could see that they were inserting drips and there were also some pipes inserted but another man told me to sit down and not peep and I did sit down*. *[GOOD; NA; Neutral*]

Others didn’t like needles and so were happy to wait outside, or found it very uncomfortable watching procedures on their child.

*I don’t like needles and nails and things like that*. *I rather waited until they were finished*. *[FAIR; PA; Positive*]

*So I walked out because I couldn’t handle seeing them putting a needle into his body*. *[POOR; PA; Positive*]

*I didn’t want to look*. *My stomach was turning; I was nauseous–like that*. *I couldn’t… because the blood was flowing out of his ear*. *[FAIR; PA; Positive*]

*It wasn’t a nice experience*. *Whenever I lie down*, *I think about what happened to him–how I saw everything; I saw how my child was suffering*. *[POOR; PA; Neutral*]

Communication and regular updates during the resuscitation were not common. In the two cases where it occurred, it seemed to improve the caregivers’ experience of the care (and maybe even negated their desire to be physically present during the resuscitation).

*… the seccy (secretary) kept… the seccy*, *right*? … *kept coming out to tell me*, *“They’re still busy with her*. *They’re still busy with her*.*” Okay*, *then she would go in and come out again*. *And that’s how it went the whole time until they came to say she didn’t make it*. *[FAIR; NA; Positive*]

*Yes*, *I felt very comfortable because they went into detail*. *When they did something*, *they would say*: *“We’re going to do this because this is for that*.*”–like that*. *[FAIR; PA; Positive*]

#### Professionalism when breaking news

There were many examples of breaking the news of death to families in what caregivers regarded as a professional manner—being called into a private room, a doctor breaking the news gently, and meaningful explanations of the cause of death without laying blame on the family.

*Whilst still waiting my brother and sister arrived*. *Whilst sitting with me we were all called in*. *The doctor then told us that when I arrived with the baby he was already in coma*, *and he has tried to assist him with his breathing*, *and inserted a drip but he could not make it*. *The baby has passed away [GOOD; NA; Neutral*]

However, some were told little or nothing about the death.

*Then they came to me and said*, *“She’s gone now*.*” And then they all walked away from me… And I sat there and then I called my mother and them…*. *But they didn’t tell me the reason why she… –nothing like that*. *As in… “Mummy*, *this is the reason…” things like that… He didn’t actually talk to me… That’s why I say*, *I really don’t know… I went in (to hospital) because my child was vomiting and she wasn’t feeling well–that’s all*. *But they didn’t tell me the reason why she… –nothing like that*. *He didn’t actually speak to me*. *[POOR; AVOID; Negative*]

#### Cause of death explained/understood:

The cause of death was not well understood in general. Reasons for this were mixed: some were never told; some recalled that they were told but they were too distressed/confused to really take in the information at the time.

*No*, *they just told me that he has already passed away and they never said what was wrong with him*. *[FAIR*, *NA; Positive*]

*I asked him what was going on and he said*, *they said he had an infection and a fever and I don’t know what else*. *I could hear him but I wasn’t listening*. *[POOR; PA; Neutral*]

Attempts to ask again or follow up about the cause of death varied: some made no further attempt, others were expectant of autopsy results or some sort of follow up and outcome of the cause of death.

*They never told me what the cause of death was*. *I do not even know what is written in her death certificate*. *[FAIR; PA; Negative*]

*But the doctor didn’t … didn’t tell me and I also didn’t ask because it… ooh*, *I was like… someone who didn’t know what was going on around her*. *I was… ooh*, *I can’t tell you… I was… But if you get the folder now*, *it should be written in there because I… honestly… When I… when the doctor told me*, *it was like I didn’t know where I was*. *[POOR; PA; Negative*]

*then they told me to wait for a post-mortem just to confirm what is the cause of death*. *They wanted to check if its food or what because she never got sick … so I am still waiting for the results because they told me that their post-mortem takes two months* … *[POOR; PA; Neutral*]

#### Mixed methods results

As shown in Tables 3 and 4, the children of four caregivers who had very negative experiences were actually assessed as having received a fair clinical quality of care. All four were cases with a poor prognosis, regardless of treatment, and it was deemed that nothing else could have been done to save the children, though there were elements of delay and some referral issues noted. However, caregivers felt they weren’t seen rapidly enough, and felt that more could and should have been done when healthcare workers decided to terminate the resuscitation. Clearly the process and communication as perceived by the caregiver was inadequate.

**Table 3:** Mixed methods outcomes (number of deaths).

		Qualitative Judgement^a^		
		Positive	Neutral	Negative
**Quantitative Assessment**^**b**^	**Good**	4	1	0
	**Fair**	8	1	4
	**Poor**	1	4	6

^a^ Qualitative Judgement is based on the caregivers’ feedback

^b^ Quantitative Assessment is based on expert assessment of the case

**Table 4:** Comparison of quantitative and qualitative assessment for each death.

Site of death^a^	Expert Panel Assessment		Qualitative Judgement^c^	Cause of Death	
	Global Assessment^b^	Avoidability of Death	Patient Satisfaction Overview	Type	Diagnosis
C	Good	Not Avoidable	Positive	Trauma	Trauma
R	Good	Not Avoidable	Positive	Medical	Cardiac
R	Good	Not Avoidable	Positive	Trauma	Trauma
R	Good	Not Avoidable	Positive	Medical	Respiratory
C	Good	Not Avoidable	Neutral	Trauma	Trauma
R	Fair	Not Avoidable	Positive	Trauma	Trauma
C	Fair	Not Avoidable	Positive	Medical	Other
C	Fair	Not Avoidable	Positive	Medical	Other
C	Fair	Potentially Avoidable	Positive	Medical	Sepsis
R	Fair	Potentially Avoidable	Positive	Trauma	Trauma
R	Fair	Not Avoidable	Positive	Trauma	Trauma
R	Fair	Potentially Avoidable	Positive	Medical	Respiratory
R	Fair	Potentially Avoidable	Positive	Medical	Cardiac
C	Fair	Potentially Avoidable	Neutral	Medical	Other
C	Fair	Potentially Avoidable	Negative	Medical	Gastroenteritis
C	Fair	Not Avoidable	Negative	Medical	Other
R	Fair	Not Avoidable	Negative	Trauma	Trauma
R	Fair	Not Avoidable	Negative	Trauma	Trauma
C	Poor	Avoidable	Positive	Medical	Respiratory
C	Poor	Avoidable	Neutral	Medical	Gastroenteritis
R	Poor	Potentially Avoidable	Neutral	Medical	Sepsis
C	Poor	Potentially Avoidable	Neutral	Medical	Sepsis
C	Poor	Potentially Avoidable	Neutral	Medical	Respiratory
C	Poor	Potentially Avoidable	Negative	Medical	Other
C	Poor	Potentially Avoidable	Negative	Medical	Other
C	Poor	Potentially Avoidable	Negative	Medical	Other
R	Poor	Potentially Avoidable	Negative	Medical	Sepsis
R	Poor	Not Avoidable	Negative	Medical	Cardiac
R	Poor	Avoidable	Negative	Medical	Respiratory

^a^ C, death outside of tertiary hospital; R, death in Red Cross War Memorial Children’s Hospital

^b^ global assessment referred to the entire referral pathway (although there may have been variable assessments at individual facilities/ transfers)

^c^ judgement of caregiver’s overall satisfaction with healthcare from interview transcript analysis only

One caregiver seemed to have had a relatively positive experience, yet was assessed as having received poor quality of care. This was an older child with a severe asthma attack, badly treated from a medical perspective, with delays, indecision and errors across two facilities, yet the caregiver related being seen rapidly, being heard and having good communication with several different providers that left her feeling positive, apparently unaware of the inadequacies of care.

*He really took a good care of him*, *he told another person there to stop what she was doing*. *[POOR; AVOID; Positive*]

However the majority of quantitative and qualitative assessments were concordant, as were the caregivers’ responses when asked if there was anything that could have been done better. There did not seem to be specific correlations to link any assessed theme (quantitative or qualitative) in terms of maternal age, delay to onset, number of steps in the pathway, or even whether caregivers were present at the final resuscitation. There were more dissatisfied caregivers of children with medical illness as opposed to trauma.

## Discussion

Our results show quite clearly that in this setting the caregivers’ experience is not necessarily related to the clinically assessed quality of clinical care delivered. We identified the main themes which caregivers related from both positive and negative perspectives and how they seem to have influenced the overall experience of their child’s death, regardless of the clinical quality of care. In most cases there was some association between the clinical assessment and the caregiver’s experience, in that there were more unavoidable deaths amongst the group with positive perceptions, and more avoidable or potentially avoidable deaths in the group with negative perceptions. But paradoxically in a few cases we see evidence of what medical experts deemed poor care, with an avoidable death, yet the caregiver appeared satisfied with the care they received and had accepted the death. This is perhaps due to some element of perceived care or communication by the healthcare team that was not captured by the research, perhaps a culturally mediated phenomenon, or the result of a complex power dynamic in the patient-health care worker dynamic. This is in accordance with other research which suggest there is a complex process of developing meaning to grieving and death in mothers, especially in a multi-cultural context such as in Cape Town [20–23].

### Family perception of effort

Triage is an effective, well implemented strategy that has been adopted by most health facilities, as evidenced by the prompt treatment received and the perceptions of caregivers. Many caregivers in this study anticipated (and accepted) delays and waiting as part of the healthcare experience, to the extent that any sense of urgency and rapid care was memorable and remarked upon. In contrast, caregivers seldom mentioned lack of care and urgency (as well as lack of empathy) which perhaps demonstrated their low expectations. The quantitative results did show profound delays (median delay from first presentation to PICU admission 12.3 hours), even unexpected ones such as at the tertiary hospital (median delay from arrival at the tertiary hospital to PICU admission of 5.0 hours), and there is clearly much room for improvement across the system despite the evidence for some well-intentioned individual clinician urgency [14].

There was a marked distinction in different caregivers’ acceptance or closure around the death of their children. Some caregivers seemed to accept the death according to their own expectations of whether the injury or illness was survivable, while those with least closure blamed delays and errors in the healthcare system. Although many caregivers desperately wanted referral of their child to a tertiary hospital, and saw this as the preferred outcome, our small sample does not show any particular association between caregiver satisfaction and the children who died at peripheral sites versus RCWMCH.

### Family presence

There is substantial research and evidence from other countries looking at the process of dealing with a child and the family of a child who dies suddenly in a healthcare facility, including whether the family is allowed or encouraged to be present during the final resuscitation [24–29]. There is sufficient evidence to say that some (most) parents would prefer to be present with their child during resuscitations (as borne out by the majority of caregivers in this study) and this is likely to aid their acceptance and coping with the death. Many healthcare workers are uncomfortable with family presence and discourage it, but our results show that caregivers are clear on their desire and would like the opportunity to be present [26, 28–30]. Yet when given the opportunity to stay with their child during invasive procedures, several parents expressed their discomfort at witnessing this, and may in fact have been expressing a felt need to be with their child rather than the reality of observing the resuscitation. This is an important point for healthcare workers who should consider the benefit of parental presence in any resuscitation–not only from a clinical perspective as caregivers often provide crucial clinically beneficial parts of the history, but also given the impact on the caregiver’s experience as we can seldom predict when a terminal resuscitation will occur. As other researchers have pointed out, caregivers in this situation need to be supported by a staff member, able to explain the procedures, liaise with the clinicians, and individualize the caregiver’s experience [28].

### Communicating and supporting caregivers when their child dies

The key needs of families are communication, empathy, and concern which are often missing in burnt out health care professionals [13]. Work by Brysiewicz conducted in a South African setting not unlike that of this study, has suggested a model to deal with sudden death in the emergency unit–encompassing planning, resources and culture before the event, optimal care of the patient and family during the death, and assistance not only to the family after the death, but also to the health care professionals involved [31]. Ganca et al in Cape Town [32] and Hassankhani et al. in Iran [33], point out the specific communications skills required by health care professionals in this situation. Furthermore, cross cultural interactions add additional challenges and require specific skills that are not necessarily part of an emergency clinician’s skill set, although desirable to deliver high-quality care [34].

Breaking the news of death to family members is always an uncomfortable and stressful procedure for healthcare workers, and even more so when a child is involved [21, 27, 35]. While our data shows some examples of good practice, such as regular and open communications with caregivers from a senior clinician, it also highlights that many frontline clinicians avoid formally and professionally breaking the news to family members, and instead break the news informally at the bedside or in a working environment, and do it hurriedly, often using euphemisms and with little further explanation or confirmation of understanding to the family. Although this must be seen in the context of a resource strained healthcare system, with health care workers stretched for time to spend with family during and after a resuscitation, it nevertheless may point to a lack of knowledge and the need for inclusion in training curricula as suggested by a recent survey of healthcare workers in Cape Town [32], as well as the need for clear policies and protocols for such times. Follow up support and communication with grieving parents needs to be part of the continuity of care following such a death, perhaps through community health and support services.

O’Malley et al refer to a “good death” which encompasses acknowledging that not all deaths are avoidable; caring for survivors (by retaining trust, giving them some control and decision making, and allowing presence and “goodbyes”); and having a clear protocol in place [36]. There were no policies in place, even in the tertiary RCWMCH for supporting caregivers. Most deaths occur outside of office hours, and in this system that usually means without the presence of senior clinicians, consultants, or any immediately available counselling service, which needs to be taken into account. Beyond palliative care, and tertiary intensive care settings, the death of a child is a rare event, and most healthcare workers will only occasionally be involved, usually unexpectedly, and it occurs more often after-hours with limited support resources in place. This is a challenge both in terms of educating health care workers, and making specific resources such as senior staff and counsellors available.

### Strengths & limitations

This study is unique in including a significant number of sudden and unexpected deaths of children, in a setting where reliable data collection is possible, and in being able to correlate biomedical assessment of the quality of care as well as the caregiver’s perception and experience.

Limitations include the differences between the three interviewers and the depth of information they were able to obtain from caregivers, as well as the cultural difference in caregivers’ expectations and willingness to share their experiences of the health care system which were not explored and differentiated. All caregivers were interviewed within two weeks of the death of their child, but clearly their bereavement processing and the circumstances around the death may have influenced their recall, and their perception of the entire process is likely clouded by the terminal event. Although just mothers were present for most interviews, the father was also present in two of the interviews which may have affected the mother’s responses and this was not analysed, yet did not seem to be the case from the transcripts. The meaning, and some expressions (as well as non-verbal cues) may have been lost in the translation (such as euphemism for death and dying), although having the interviewers translate the transcripts themselves soon after the interview helped to mitigate this. Having the transcripts translated by the interviewer may have introduced bias, but they were all experienced researchers with no involvement in any aspect of the study other than conducting their own interviews. Due to our sampling, there were few neonatal deaths which is not representative–South African literature suggests almost half of childhood deaths are in the neonatal period [1]. And the assessment of the quality of care used was a global one for the entire referral process which may have entailed steps of varying quality, perhaps skewed by the places where major events took place (as would be the case for the caregivers’ experience).

## Conclusion & recommendations

In this study, which represents a significant number of childhood deaths over a year long period across a spectrum of healthcare facilities and presentations, we show there is considerable discrepancy between different measures of quality of care. 40% of this cohort were assessed to have had clinically poor care and 45% of these deaths were assessed as either avoidable or potentially avoidable. It should therefore be no surprise that around half of the caregivers were unimpressed by the care delivered. However, the drivers behind caregivers’ perceptions of quality of care are different from the criteria used to determine clinical quality of care, as highlighted by the discordant cases. Recognising this discrepancy and attending to those features which impact caregivers’ experiences could have enormous effect on the families of children who die suddenly, and likely on the clinicians who manage such cases. Relatively simple and high value interventions could be adopted to address many of these, such as better training of healthcare workers in dealing with death and dying in emergency healthcare settings, policies and protocols in place for how to break the news and support bereaved family (including systems that work after-hours), follow up with families after a death to offer ongoing support and counselling and creating spaces in institutions to talk to families. Processes to routinely investigate deaths and consider underlying factors whilst in place, might be expanded to include an evaluation of the family’s experience of care, and those factors which most affected their perceptions of the service they received.

## Supporting information

S1 Table Interview guide. (DOCX)
