# Supplementary material for: “She’s gone now.” A mixed methods analysis of the experiences and perceptions around the deaths of children who died unexpectedly in health care facilities in Cape Town, South Africa
Source: PLoS One. 2019 Mar 6;14(3):e0213455. doi: 10.1371/journal.pone.0213455 (PMC6402763; doi:10.1371/journal.pone.0213455)
Supplement: S1 Table — (DOCX) [file pone.0213455.s001.docx]

## Supplementary Table 1: Caregiver Interview Schedule

**DEMOGRAPHIC DATA:**

1. Name of Child

2. Date of Birth

3. Relation with Interviewee

4. Address (where child resides)

5. Name of Mother

Age

Marital Status

Language

Highest educational level

Employment Status

Contact no.

Contact Employer (if employed)

6. Biological Father

Age

Marital Status

Address (if different from mother and child)

Language

Highest educational level

Employment Status

Contact no.

Contact Employer (if employed)

7. Primary care giver (if not biological mother)

Name

Age

Address

Contact no.

Marital Status

Relation to child

**SOCIAL DATA**

1. Name of Residential Area

2. Description of Area/locality: Informal Formal Other, specify

3. Type of Dwelling: Formal Informal Traditional Other, specify.

4. Construction of Home

5. Number of Rooms

6. Amenities (water/ electr/ sewage/ etc.)

7. Assets

8. Nearest Health Facility:

Name of Facility

Distance from your House

Hours of Service at Facility

Mode of transport to nearest clinic

9. Nearest 24hr service Health Facility (If same as 4 above, skip and go to 6)

Name of Health Facility

Distance from Home

Mode of transport to 24hr health Facility:

10. Ambulance services:

What do you do if:

If your child is very sick at home?

Your child is involved in an accident?

Why do you not call an ambulance?

• Do not know the telephone number

• Do not have money to pay for ambulance

• It does not come into my community

• Other, specify

11. Financial Support:

Household Income

Receiving any Grant: Yes … No .…if yes, type of grant

Is Father maintaining the child: Yes ….… No ….

If Not, is he paying Child Maintenance: Yes No

12. Family Data:

How many children are currently directly dependant on you? …

Gender of each child Multiple Birth: Y/N Age of child Alive: Y/N

Have any children died while under your care?

**CARE GIVEN AT EACH FACILITY:**

1. Can you tell me what happened to your child from the onset of the illness i.e. the first signs of illness in your child?

1.1.1. What were the signs?

1.1.2. How long ago did these signs begin? (note date/time)

1.1.3. When did you decide to seek medical help?

1.1.4. What made you decide to seek medical help? (note date/time)

1.1.5. Before seeking medical help, did your child receive any treatment for this illness? If so:

1.1.5.1. Can you please describe the treatment?.

1.1.5.2. Who provided the treatment (caregiver, family, traditional healer, chemist, other – note name of helper and facility) .

1.1.5.3. In your opinion, was the treatment helpful?

*If the child is SICK, ask question a) MEDICAL below:*

*If the child has been injured, accident etc., skip and ask b)TRAUMA below:*

a) **MEDICAL CASE**

2. Tell me about all the steps that you followed in seeking care for your child, distance travelled, mode of transport and costs, time of arrival at each facility, waiting time at each facility, when were you attended to and your perception of care provided.

2.1. Name of Facility visited

2.2. Time you left home …Mode of Transport Cost/single trip …

2.3. Time of Arrival at facility …

2.4. What was the first thing that was done upon entering the facility (checking of vital signs/ triage)

2.5. Was there a separate queue for children and adults? Yes No

2.6. Time of being attended by a health care provider

2.7. In the case of Gastro:

2.7.1. Was the child given any oral hydration? Yes… No…For how long: ….

2.7.2. Was a drip put up? Yes . No If yes, when

and where who put it up? Doctor …. Nurse …

2.8. In the case of ASTHMA or Respiratory Distress:

2.8.1. Was the child nebulised? Yes .No if yes, when …

2.8.2. Was the process repeated? Yes … No

2.9. In the case of FEVER or SEPTIC SHOCK:

2.9.1. Was the child’s urine tested? Yes . No … at what time …

2.9.2. Were you told why the urine is being tested? Yes No .

2.10. In the case of Convulsions

2.10.1. How frequent were the convulsions whilst you were still at home? …

2.10.2. If you called an ambulance, what was done when the ambulance arrived .

If not, on arrival at the facility?

2.11. Do you feel that you were attended quite quickly: Yes… No and why do you say so?

2.12. What was the outcome

2.13. What were you told about your child’s illness

2.14. Who explained the child’s illness to you?

2.15. In your opinion, could the medical care provided to your child have been improved?

If so, how?.

b) **TRAUMA PATIENT**: (Scene of accident)

2. What time did the accident/injury occur? .

2.3. What was the first assistance you received?

2.4. Who was with you at the time ….

2.5. Was any first aid care done? Yes… No if yes, by whom?

2.5.1.1. What was done ….

2.6. Was an ambulance called? Yes No if yes, by whom?

2.7. When did the ambulance arrive? How long was the waiting? Short/long. Estimate period of waiting …

2.8. How long did it take you to get to the nearest Health Care Facility from the scene of the accident?

2.9. What care was given to your child in the ambulance, on the way to the nearest health facility?

2.10. What injuries did the child sustain? …

2.11. Was the extent of the injuries explained to you at the scene of the accident?

2.12. What was the level of consciousness of the child at the scene of accident?

2.12.1.1.1. Alert React to voice React to pain Unresponsive .

2.13. Were you (parent/carer) allowed to stay with the child throughout the time? Yes No

2.14. Name of Facility visited …

2.15. Time you left accident scene …Mode of Transport Cost/single trip …

2.16. Time of Arrival at Health facility …

2.17. Who directed you about where to go in the facility?

2.18. What was the first thing that was done upon entering the facility?

2.18.1.1.1.1. (checking of vital signs and triage)

2.19. Was there a separate queue for children and adults?

2.19.1.1.1. Yes No

2.20. Time of being attended by a health care provider

2.21. Do you feel that you were attended quite quickly: Yes… No…. and why do you say so?

2.22. What was the outcome ?

2.23. What were you told about your child’s illness

2.24. Who explained the child’s illness to you?

2.25. In your opinion, could the medical care provided to your child have been improved?

2.25.1.1.1.1. If so, how?.

3. Please tell me about anything you feel is important that was not covered in the interview…

**INTERVIEW FEEDBACK**

1. How stressful did you find taking part in this interview? (tick one)

Not at all A little Somewhat Very stressful

2. How did you find the length of the interview?

About all right Too long Too short

3. Would you be prepared to participate in this type of interview again?

Yes No
